# The combined anticancer of peanut skin procyanidins and resveratrol to CACO‐2 colorectal cancer cells

**DOI:** 10.1002/fsn3.3590

**Published:** 2023-08-03

**Authors:** Na Wang, Enguang Gao, Chenxu Cui, Fan Wang, Hongtao Ren, Chao Xu, Cancan Ning, Yuru Zheng, Qingqing Liu, Qiuying Yu, Gaiping Zhang

**Affiliations:** ^1^ College of Food Science and Technology Henan Agricultural University Zhengzhou China; ^2^ College of Animal Medicine Henan Agricultural University Zhengzhou China; ^3^ Key Laboratory of Nutrition and Healthy Food of Zhengzhou Zhengzhou China; ^4^ International Joint Research Center for Animal Immunology Zhengzhou China; ^5^ Longhu Laboratory of Advanced Immunology Zhengzhou China; ^6^ School of Advanced Agricultural Sciences Peking University Beijing China

**Keywords:** colorectal cancer, procyanidins, resveratrol, signaling pathway, synergistic anticancer

## Abstract

Colorectal cancer is one of the leading causes of cancer deaths worldwide. Currently, chemotherapy is the primary way for colorectal cancer, but with severe side effects. Therefore, it is urgent to find safer and more effective adjuvant treatment methods. At present, natural active substances are promising alternatives, as numerous studies have demonstrated possible synergistic anticancer effects in plant‐active polyphenols. In the present study, the combined effect of procyanidins (PC) (from peanut skin) and resveratrol (RES) (from peanut buds) on the synergistic anticancer potential was investigated. CACO‐2 and HCT‐8 cells were served as colorectal cancer models, and HEPG‐2 and HUH‐7 cells were served as liver cancer models to observe the effects of PC and RES alone or in combination on the growth and proliferation of these four types of cancer cells. The results revealed that both PC and RES could inhibit the cells’ proliferation in a manner with concentration‐dependent, but they exerted synergistic anticancer effects only on CACO‐2 cells. PC and RES could synergistically inhibit CACO‐2 cell clone formation, inducing apoptosis of CACO‐2 cells and blocking their cell cycle in G0/G1 phase. Additionally, as observed by the results of Western blot assay, the combined effect of PC and RES also inhibited the phosphorylation of Thr308, Ser473, and ERK and promoted the phosphorylation of IKBα and NF‐κB in CACO‐2 cells. These findings collectively indicate that PC combined with RES might exert synergistic anticancer effects by regulating AKT, ERK, and NF‐κB signaling pathways.

## Graphical abstract

In the present study, the synergistic anticancer potential of procyanidins (PC) and resveratrol (RES) extracted from peanut skin and peanut buds, respectively, was investigated, when applied in combination. CACO‐2 and HCT‐8 cells as colorectal cancer models and HEPG‐2 and HUH‐7 cells as liver cancer models were used to observe the effects of PC and RES alone or in combination on the growth and proliferation of these four types of cancer cells.

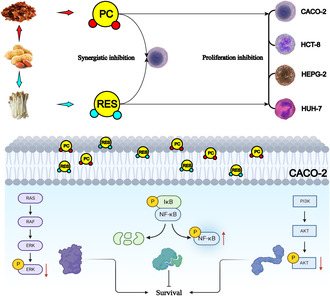

## 1 INTRODUCTION

Colorectal cancer is the third most prevalent malignancy in the world and one of the leading causes of death from cancer worldwide (Bray et al., 2018; Collaborators, 2022). Although the rapid advances in cancer treatment over the past decade, most colorectal cancer patients tend to have poor outcomes because they were already in the middle to late stages when they were diagnosed (Cao et al., 2020). Currently, the therapy for colorectal cancer is mainly based on chemotherapy, but accompanied by many side effects, such as iron deficiency anemia, nausea and vomiting after chemotherapy, etc. (Zraik & Hess‐Busch, 2021). Breast cancer patients treated with paclitaxel chemotherapy experience long‐term peripheral neuropathy (Chiu et al., 2022). These negative effects emphasized the urgency of exploring new approaches to achieve lower side effects and more effective adjuvant treatment of colorectal cancer.

In recent years, numerous studies were conducted on the anticancer effects of polyphenolic compounds, widely presented in various nuts, vegetables, and fruits. Most of the polyphenolic compounds, such as procyanidins, resveratrol, and curcumin, have contributed to the strong antioxidant properties and were proved to inhibit the growth and various cancer cell proliferations through multiple pathways. Researchers have reported that curcumin could inhibit the proliferation and metastasis of TNBC cells through the Hedgehog/Gli1 signaling pathway. Resveratrol caused the A549 cells’ death through the STAT‐3 signaling pathway. Polyphenol procyanidin‐b2 inhibited hepatocellular carcinoma cell proliferation and hepatocellular carcinogenesis through direct binding and inhibition of AKT activity (Li et al., 2022; Li et al., 2016; Liu, Shi, Wang, Li, He, et al., 2020; Prasad & Katiyar, 2013; Yang et al., 2018; Zhang et al., 2013; Zhang et al., 2018). Various polyphenolic compounds have different anticancer mechanisms, and thus the combined application of multiple natural compounds may result in synergistic anticancer effects. Previous studies have proved that the combination of resveratrol and EGCG could induce synergistic apoptosis and inhibit SCCHN xenograft growth in vivo (Amin et al., 2021), and the combined effect has also been demonstrated in both resveratrol and rapamycin. The integrated impact of rapamycin and resveratrol had a synergistic effect in inhibiting the papillary thyroid cancer proliferation, invasion/migration cells, and apoptosis, compared to rapamycin and resveratrol alone (P. Bian et al., 2020). Additionally, both the resveratrol and rapamycin together could induce cell death, specifically in human bladder cancer cell lines. A growing number of synergistic anticancer studies have exhibited the potential advantage in mixtures of multiple polyphenolic compounds for the therapy of different types of cancers. However, there is a limited understanding regarding the synergistic anticancer effect of Procyanidins from peanut skin (PC) in combination with resveratrol extracted from peanut buds (RES).

The procyanidins are abundant in the peanut skin. Studies have reported that procyanidins accounted for 17% of the total mass of mature peanut skin, 50% of which were oligomers with high biological activity (Karchesy & Hemingway, 1986). The constituent monomers of peanut skin procyanidins are (+)‐catechin and (−)‐epicatechin, a low degree of polymerization with an average of 3.2 (Gu et al., 2003; Lazarus et al., 1999; Yu et al., 2006), in which the monomer content is low and the dimer content is high, mainly for Procyanidin A. Procyanidin A are dimeric forms of procyanidins that link two monomers in an epoxy form. Numerous studies have demonstrated that type A procyanidins have multiple biological functions, including anti‐inflammatory (Dudek et al., 2017), antioxidant, antiviral (Xu et al., 2010), and anticancer (Vilkickyte et al., 2020).

Resveratrol is a non‐flavonoid polyphenolic compound that is widely found in peanuts, grapes, thuja, and other plants (Koushki et al., 2018), characterizing with antioxidant, anti‐inflammatory, and anti‐tumor. The results of numerous studies have shown that resveratrol can play a chemopreventive role in the development, progression, and metastasis stages of tumor diseases such as liver, colorectal, prostate, and breast cancers (Ren et al., 2021).

It is generally accepted that using the natural active substances of “medicinal and edible” as drugs is safer than traditional chemically synthesized drugs. Therefore, it is necessary to maximize the effects of promising anticancer drugs that have been discovered. The objectives of this study are to investigate the anticancer effect of the combined action of PC and RES, specifically as follows: (1) investigate the growth inhibitory effects of PC and RES alone or together on colorectal and liver cancer cells, and (2) CACO‐2 cells as a representative model is used to reveal the possible mechanisms of their synergistic anticancer effects by studying the effects of PC and RES alone or in combination on cell clone formation, apoptosis, cell cycle, AKT, and other signaling pathways.

## 2 MATERIALS AND METHODS

### 2.1 Antibody

The antibodies used in this study are detailed in Table 1.

**TABLE 1:** The primary antibodies used in this study.

Antibodies	Source	Dilution ratio	Cat
AKT	Signalway antibody	1:5000	48,888
p‐AKT(S473)	Signalway antibody	1:1000	11,054
p‐AKT(T308)	Signalway antibody	1:1000	11,055
ERK	Abmart	1:10000	T40071
p‐ERK	Abmart	1:2000	T40072
IKBα	Cell signaling	1:1000	4814S
p‐IKBα	Cell signaling	1:1000	9246S
NF‐κB p65	Cell signaling	1:1000	8242S
p‐NF‐κB p65	Cell signaling	1:1000	3033S
β‐Actin	Signalway antibody	1:10,000	21,338
GAPDH	Abmart	1:5000	M20006

### 2.2 Extraction of PC and RES

PC and RES were provided by Key Laboratory of Nutrition and Healthy Food of Zhengzhou. PC extraction from peanut skin was conducted according to the method of our laboratory. Peanut skin was ground, and oil was removed by petroleum ether, and then was dried. 70% ethanol was added, and peanut skin was extracted jointly by microwave and ultrasonic. The crude extract was purified by AB‐8 macroporous. Thereafter, the filtrate was filtered through a 0.22 μm filter membrane. The content of PC was 95% by high‐performance liquid chromatography (HPLC). At the same time, high‐performance liquid chromatography–tandem mass spectrometry (UPLC‐QTOF) analysis was carried out. The main components were Procyanidin A dimer, Procyanidin A trimer, Procyanidin A tetramer, Catechins, and Protocatechualdehyde, and a little of B‐type procyanidins. Then, experiments were conducted.

RES extraction from peanut sprouts was conducted based on the method of Wang (C. Wang et al., 2021). The content of RES was 84% by HPLC. At the same time, UPLC‐QTOF analysis was carried out. The main components were oxidized resveratrol, resveratrol glycosides, resveratrol dimers, and tetramers. Subsequent experiments were conducted (Table S1, Figure S1 and S2).

### 2.3 Cancer cell lines and cell culture

Human colorectal cancer cell lines (CACO‐2 and HCT‐8) and liver cancer cell lines (HEPG‐2 and HUH‐7) were purchased from ATCC. The cells were cultured in Dulbecco Minimal Essential Medium [DMEM, Invitrogen Trading Co. Ltd.) containing 10% fetal bovine serum (Sijiqing) and 1% penicillin–streptomycin in a humidified incubator (Thermo Fisher Scientific Co., Ltd.) with 5% CO_2_ at 37°C, and sub‐cultured with 0.25% trypsin–EDTA (with phenol red), when it reached 90%.

### 2.4 Cell proliferation assays

The effects of PC and RES on cancer cell proliferations were performed by CCK8 (Cell Counting Kit‐8, meilunbio). Cancer cells in their logarithmic growth phase were seeded in a 96‐well plate at a density of 9000 cells/well for 24 h. Then, the cell medium with fresh medium containing PC and RES was replaced with different concentrations for 24 h. And then, cell proliferation activity was measured at different time periods according to the instructions of the CCK‐8 kit.

### 2.5 Colony formation assay

The CACO‐2 cells were seeded into 6‐well plates (500 cells/well) for 24 h and then replaced the supernatant with fresh medium containing different concentrations of PC and RES. After 24 h of incubation, the cells were treated with fresh medium for 14 days until the formation of visible colonies. And then, colonies were washed with PBS and fixed with 4% paraformaldehyde for 30 min, stained with crystal violet for 15 min. After staining, the cells were washed with distilled water twice and colony numbers were counted.

### 2.6 Cell cycle assay

The CACO‐2 cells (5 × 10^5^ cells/well) were placed in 6‐well plates and starved with serum‐free DMEM medium for 24 h before treating the cells with PC and RES. After 12 h of sample treatment, the cells were washed twice with PBS, centrifuged at 1500 rpm for 5 min, and collected at a concentration of 1 × 10^6^/mL. After centrifugation of the prepared single cell suspension, the supernatant was removed and 500 μL of 70% precooled ethanol was added to the cells for overnight fixation. And then, 100 μL of RNase A solution was added to the cell precipitate and resuspended the cells in a water bath at 37°C for 30 min. Finally, 400 μL of PI staining solution was added and mixed well, incubating for 30 min at 4°C in the dark. Cell cycle distributions were detected by flow cytometer (Beckman Coulter), and the results were analyzed by FlowJo 10.8.1.

### 2.7 Apoptosis analysis

Annexin V‐FITC Apoptosis Detection Kit (Beyotime Biotechnology) was used to detect cell apoptosis. Briefly, the CACO‐2 cells were seeded in a 6‐well plate for 24 h and then treated with PC and RES at different concentrations for another 24 h. 50,000–100,000 resuspended cells were taken and centrifuged at 1000 g for 5 min. The supernatant was discarded, and 195 μL Annexin V‐FITC conjugate was added to gently resuspend the cells. Thereafter, it was stained with 5 μL Annexin V‐FITC and 10 μL propidium iodide (PI) for 20 min at room temperature in the dark. The percentage of apoptotic cells was analyzed using a flow cytometer.

### 2.8 Western blotting

Total proteins from CACO‐2 cells were treated with PC and RES, and their combinations were extracted using RIPA lysis buffer containing a protease and phosphatase inhibitor cocktail. Protein concentrations were quantified by BCA Protein Assay Kit (solarbio) and separated by SDS‐PAGE (10%) for electrophoresis and then transferred to a polyvinylidene difluoride (PVDF) membrane. Next, membranes were saturated with 5% non‐fat milk for 2 h at room temperature and then incubated with primary antibodies overnight at 4°C. Goat anti‐mouse and anti‐rabbit IgG conjugated with horseradish peroxidase (HRP) were used as secondary antibodies to incubate with the membranes for 1 h at room temperature. Immunoreactive bands were visualized using Chemiluminescence kit and determined by an infrared imaging system (Bio‐rad, United States). Quantitative analysis was conducted by Image J 1.53.

### 2.9 Statistical analysis

All data are given as mean results of at least three independent measurements and shown as means ± SD. The data were quantified for graphing using GraphPad Prism software v8.0.2. Where it was necessary to compare three groups of data, a one‐way ANOVA test was performed using IBM SPSS Statistics software v26. To determine significant differences between treatments, Duncan's test was employed with a significance level set at *p* < .05. Different letters represent statistically significant differences (*p* < .05).

## 3 RESULTS

### 3.1 Proliferation inhibition of PC and RES in human colorectal and liver cancer cells

**FIGURE 1:**
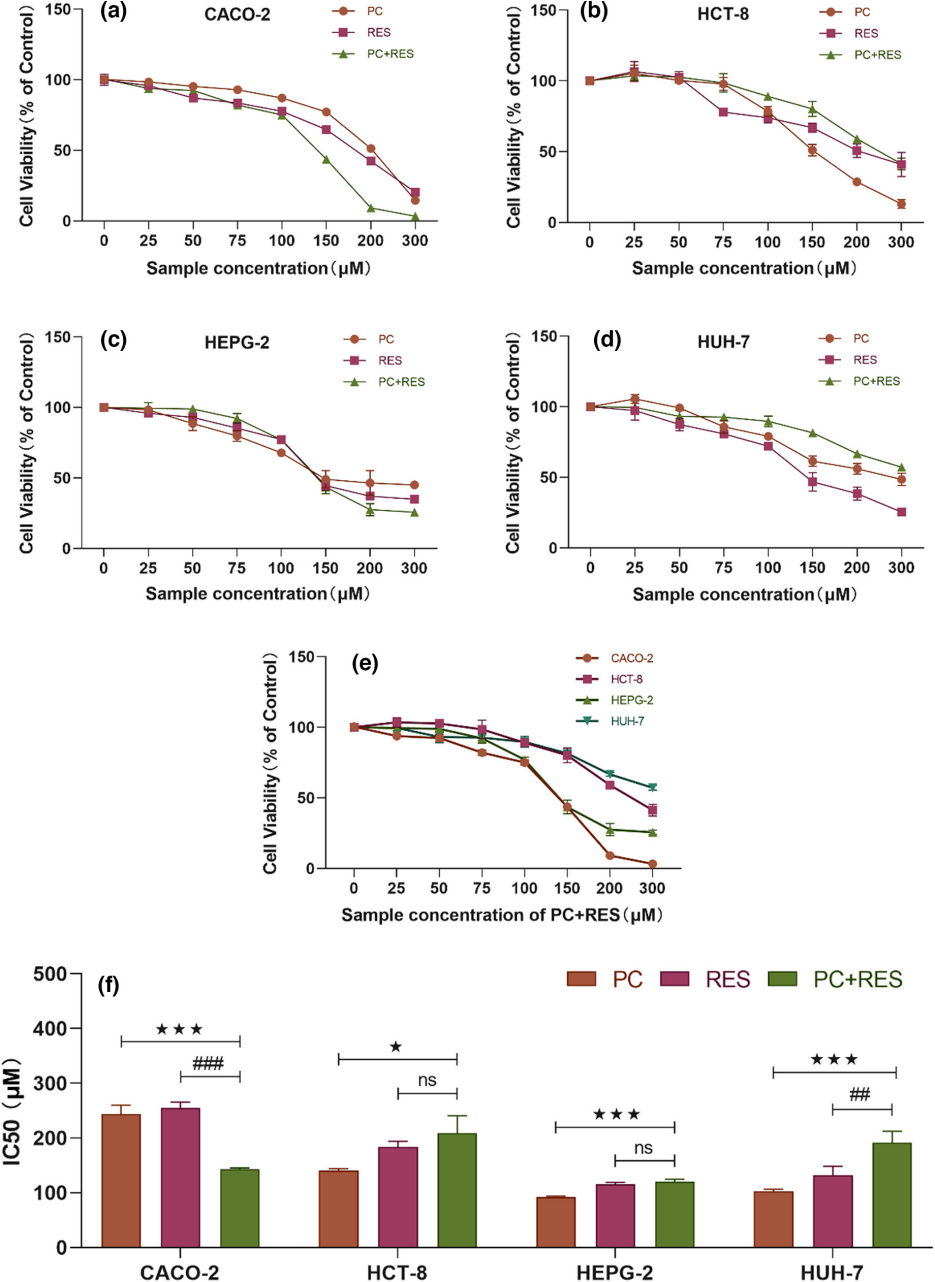
Effects of PC and RES treatment for 24 h on the proliferation of colorectal and liver cancer cells. (a) CACO‐2 cells, (b) HCT‐8 cells, (c) HEPG‐2 cells, and (d) HUH‐7 cells, (e) Four types of cells, (f) the IC50 values in four types of cells were treated with various concentrations of PC, RES, or their combination (1:1 molar concentration ratio of both samples, and the total molar concentration of the two samples is equal to the concentration of the separate treatment groups of either sample at the same concentration). *p < .05, ***p < .001 compared to PC group. ^##^ *p* < .01, ^###^ *p* < .001 compared to RES group. ^ns^ *p* > .001 compared to RES group. Control indicates the group without PC and RES.

The CCK8 assay was used to verify whether PC and RES could synergistically inhibit the growth and proliferation of human colorectal and liver cancer cells. CACO‐2, HCT‐8, HEPG‐2, and HUH‐7 cells were treated with different concentrations of PC, RES, or their combinations for 24 h in this study. Four types of cell proliferations could be inhibited by PC or RES in a concentration‐dependent manner, and only the CACO‐2 colorectal cancer cell proliferation was synergistically inhibited by PC and RES (Figure 1a–d). The IC50 values of PC, RES, and their combinations on these four cell types were analyzed using GraphPad Prism software v8.0.2 (Table 2 and Figure 1f). Their combined treatment group showed significantly lower IC50 values for CACO‐2 cells compared to the PC or RES treatment groups, suggesting that PC and RES could synergistically inhibit the growth and proliferation of CACO‐2 colorectal cancer cells, and no similar result was observed in the other three cells. Additionally, CACO‐2 was also the most sensitive cell among these four types of cells to the combined treatment of PC and RES (Figure 1e) and was selected for further study.

**TABLE 2:** Effects of PC, RES, and their combinations on the proliferation activity of cancer cells.

Cell type	Group	IC50 (μM)
CACO‐2	PC	244.13 ± 16.10^a^
	RES	254.97 ± 10.47^a^
	PC + RES	143.03 ± 2.28^b^
HCT‐8	PC	140.50 ± 3.38^b^
	RES	183.55 ± 10.26^a^
	PC + RES	208.67 ± 31.89^a^
HEPG‐2	PC	91.97 ± 2.15^b^
	RES	115.67 ± 3.41^a^
	PC + RES	120.27 ± 4.35^a^
HUH‐7	PC	103.19 ± 3.44^b^
	RES	131.80 ± 16.59^b^
	PC + RES	190.90 ± 21.19^a^

*Note*: Different superscript letters represent significant differences.

### 3.2 Clone formation of CACO‐2 cells is synergistically inhibited by PC and RES

**FIGURE 2:**
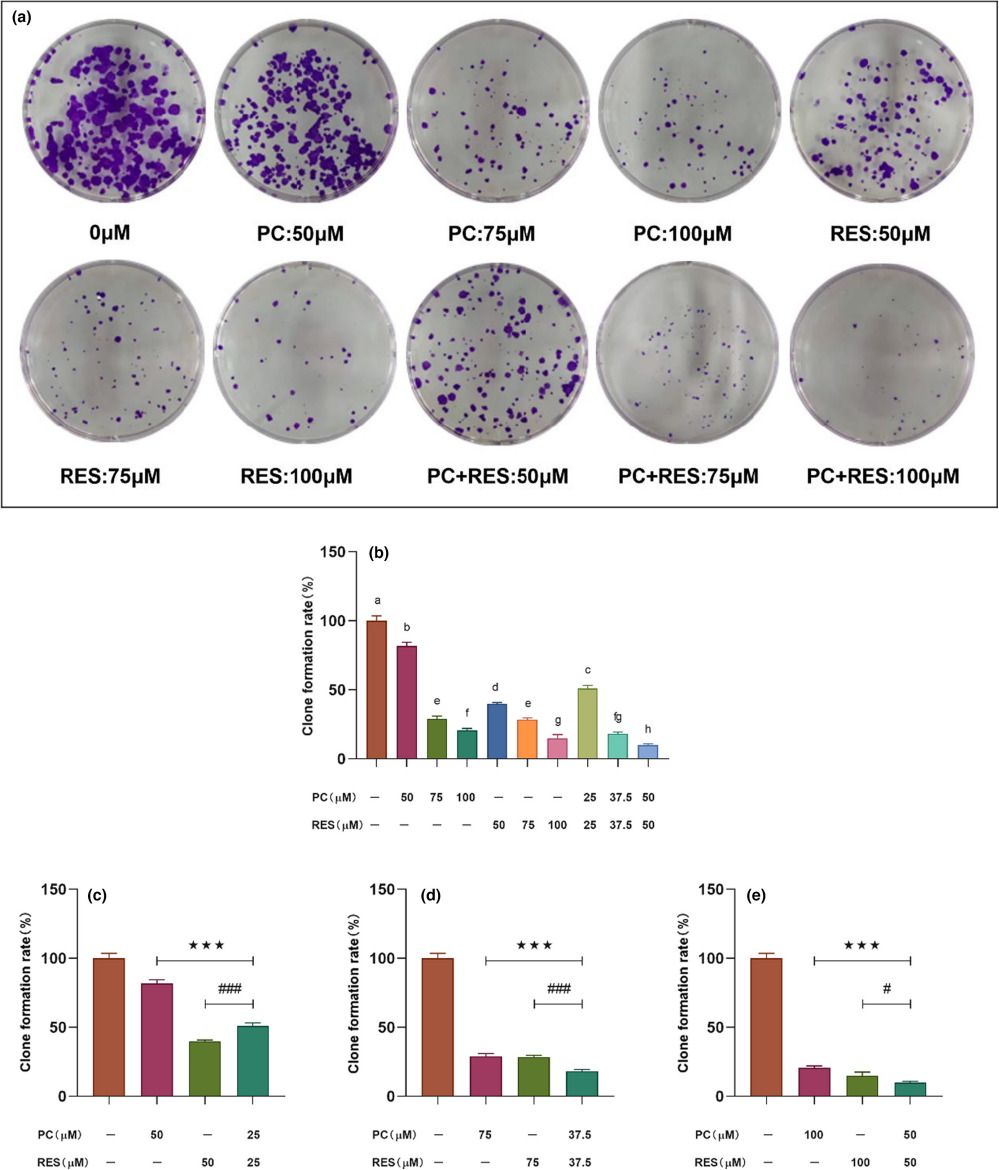
The influence of PC, RES, and their combination treatment for 24 h on CACO‐2 cell clone formation. (a) Macrograph of colony formation for CACO‐2 cells after 14 days; (b) Colony formation rate corresponding to result A. (c–e) Results of the significance analysis of the effect of the combined treatment group of PC and RES versus the treatment group alone on the clone formation of caco‐2 cells. ***p < .001 compared to PC group. ^#^ *p* < .05, ^###^ *p* < .001 compared to RES group.

Cell clone formation assay is the most effective way to evaluate cell proliferation ability, invasiveness, sensitivity to killing factors, and other items. Therefore, the cell clone formation assays were used to verify the combined effect of PC and RES on the late growth and proliferation capacity of CACO‐2 cells. As shown in Figure 2a,b, the clone formation rate in the PC, RES, and the combined treatment group decreased significantly, compared with the control group. Furthermore, the combined treatment group could facilitate inhibit the colony formation ability than other groups at the same concentrations of 75 and 100 μM (Figure 2c–e) and suggested that PC combined with RES treatment might synergistically inhibit the late development of colorectal cancer.

### 3.3 Effect of PC and RES on the cell cycle distribution of CACO‐2 cells

**FIGURE 3:**
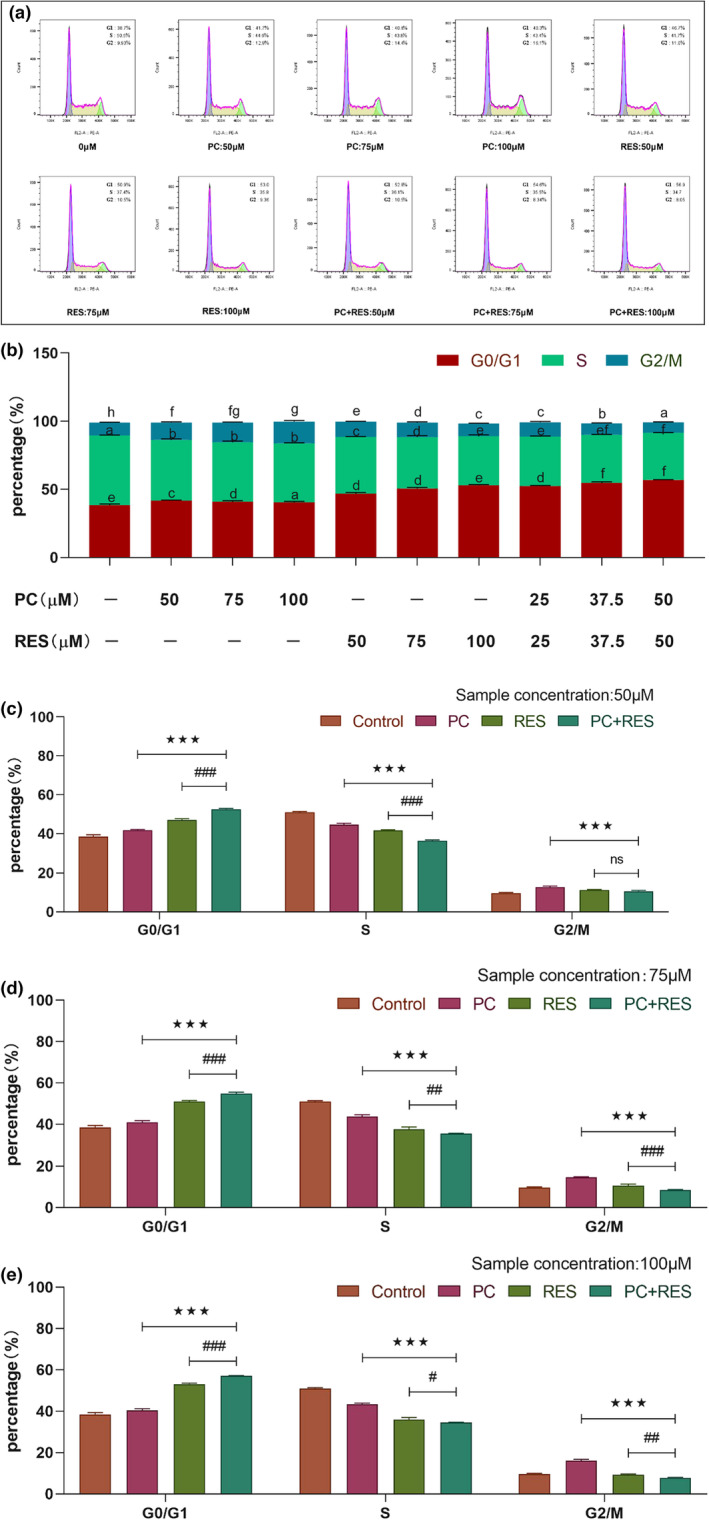
Cell cycle changes of CACO‐2 cells were measured by flow cytometry after 24 h of treatment with different samples. (a) Cell cycle distribution of CACO‐2 cells. (b) Result of cell cycle analysis in CACO‐2 cells. (c–e) Results of the significance analysis of the effect of the combined treatment group of PC and RES versus the treatment group alone on cell cycle of CACO‐2 cells. ***p < .001 compared to PC group. ^#^ *p* < .05, ^##^ *p* < .01, ^###^ *p* < .001 compared to RES group. ^ns^ *p* > .001 compared to PC or RES group.

Cell cycle dysregulation is a fundamental marker of cancer spread. To detect the effect of the samples on the cell cycle, using a flow cytometry to examine the cell cycle distribution. As the Figure 3a,b shows, the proportion of cells in G0/G1 phase was significantly higher after PC and RES treatment alone or together compared to the control group (Figure 3a,b), indicating that they were able to block CACO‐2 cell cycle in G0/G1 phase at the three concentrations. In addition, one‐way ANOVA showed that the cells’ proportion in G0/G1 phase was significantly higher in the combined treatment group than in the PC and RES treatment groups alone (Figure 3c–e), indicating that PC and RES were able to synergistically block the CACO‐2 cell cycle in G0/G1 phase.

### 3.4 CACO‐2 cells are synergistically induced to apoptosis by PC and RES

**FIGURE 4:**
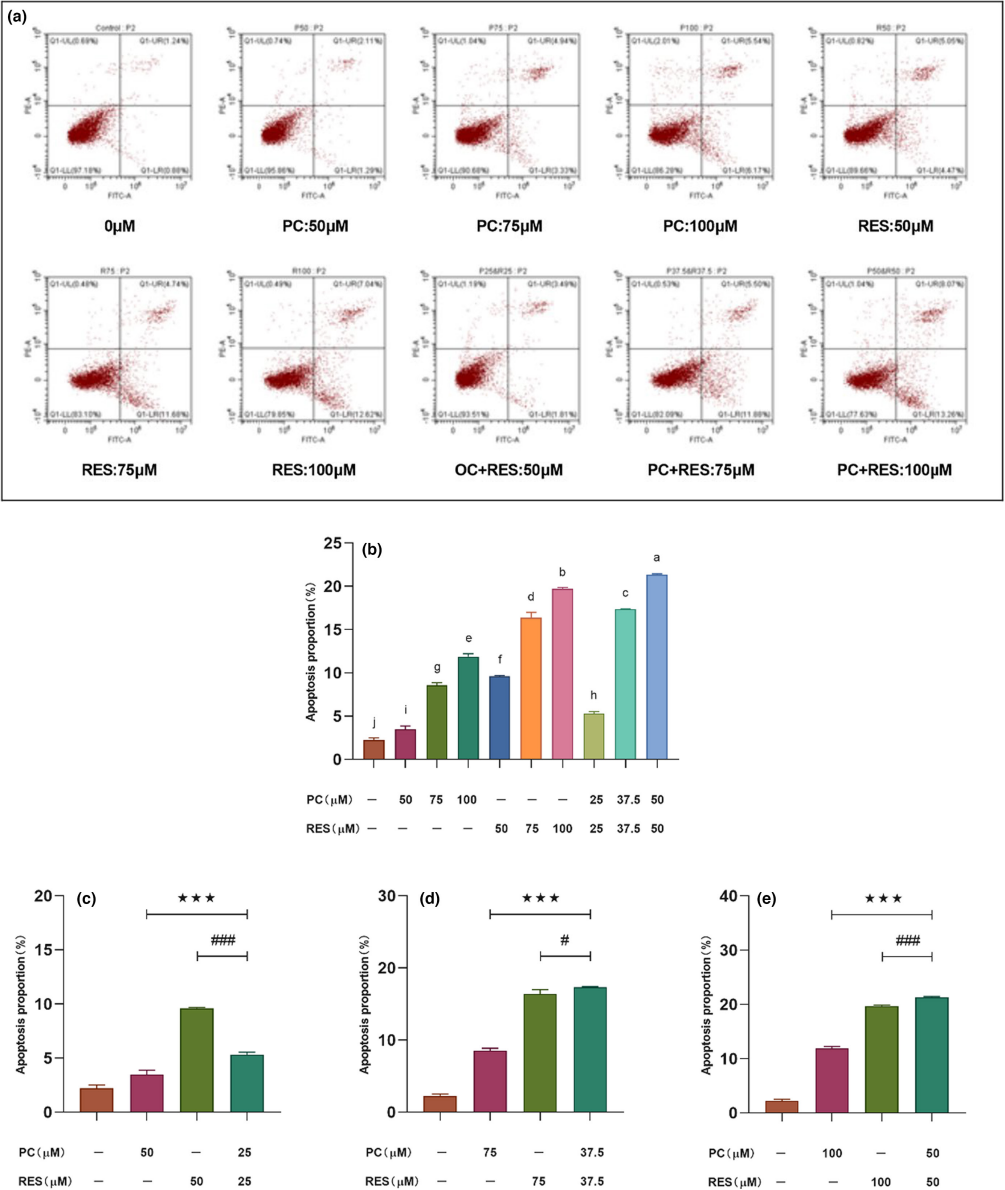
Apoptosis of CACO‐2 cells determined by flow cytometry. (a) The effect of different sample treatments for 24 h on CACO‐2 cell apoptosis. (b) Graphs with quantitative data for the apoptosis in CACO‐2 cells. (c, d, e) Results of the significance analysis of the effect of the combined treatment group of PC and RES versus the treatment group alone on apoptosis of CACO‐2 cells. ***p < .001 compared to PC group. ^#^ *p* < .05, ^###^ *p* < .001 compared to RES group.

Flow cytometry is used to detect if the samples can induce apoptosis of the CACO‐2 cells. The results showed that the apoptosis rate of CACO‐2 cells was significantly higher after treatment with PC and RES alone or in combination, which suggested that apoptosis was the main pathway for sample‐mediated cell growth decline (Figure 4a,b). After the treatment of different concentrations, the apoptotic rate of the combined treatment group was increased from 5.33% ± 0.21% to 17.37% ± 0.05%, 21.33% ± 0.13%, and the cells apoptosis was increased from 2.21% ± 0.30% to 3.49% ± 0.39%, 8.54% ± 0.33% in the PC group and from 9.60% ± 0.09% to 16.39% ± 0.59%, 19.73% ± 0.14% in the RES group, respectively. When the sample concentration was greater than 75 μM, the total apoptosis rate of the combined treatment group was significantly higher than that of the PC and RES treatment groups alone (Figure 4c–e). These results were consistent with the results of previous cytotoxicity assays and cell clone formation assays, further demonstrating that PC and RES could synergistically inhibit the growth and proliferation of CACO‐2 cells.

### 3.5 Effects of PC and RES on AKT, ERK, and NF‐κB signaling pathways in CACO‐2 cells

Numerous studies had reported that aberrant activation of various signaling pathways could regulate the proliferation and survival of various cancer cells, such as AKT, ERK, and NF‐κB signaling pathways. To investigate the mechanism of synergistic anticancer effects of PC and RES, the changes of these three signaling pathways in CACO‐2 cells were examined after intervention of PC and RES by Western blot assay.

#### 3.5.1 AKT signaling pathway in CACO‐2 cells is synergistically inhibited by PC and RES

**FIGURE 5:**
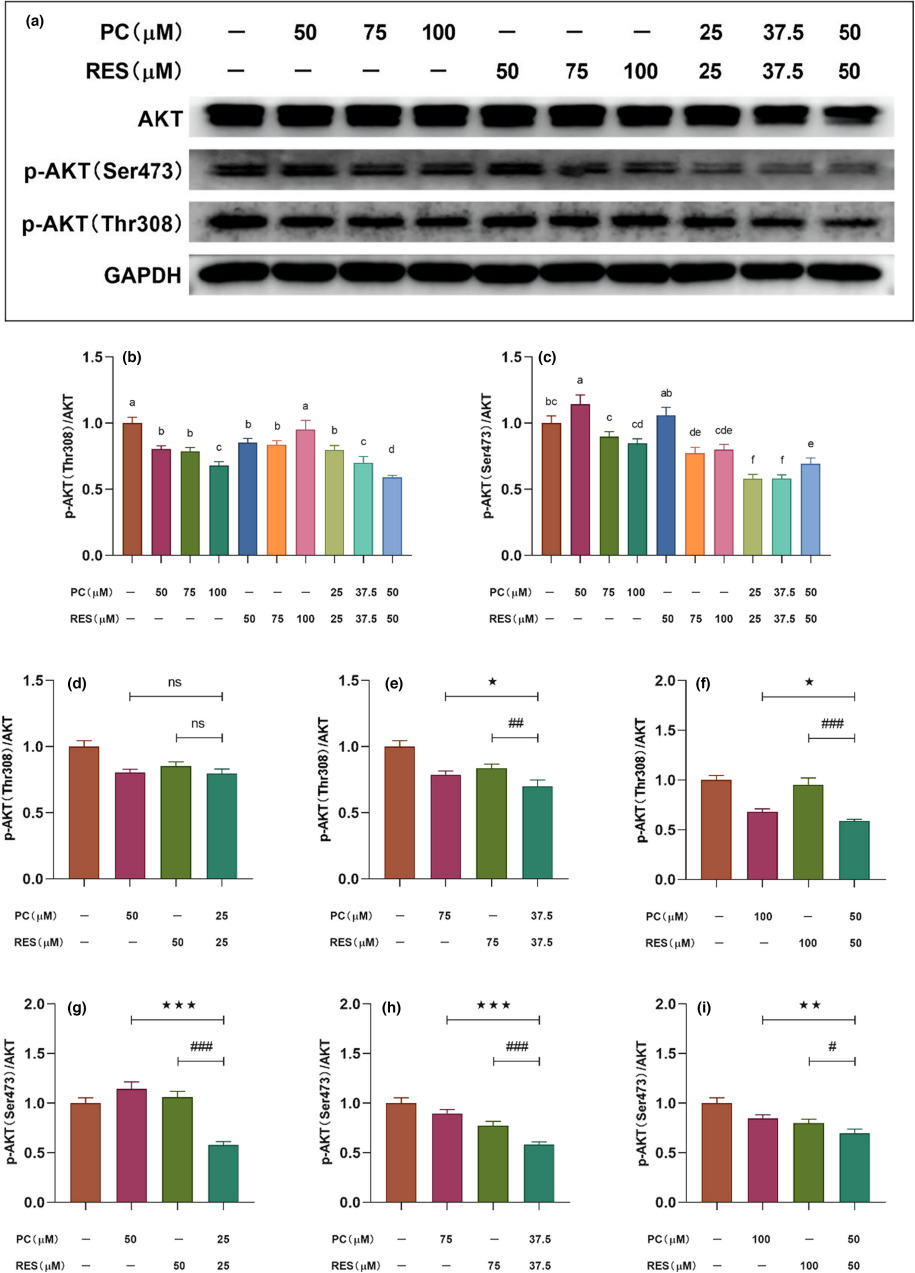
Changes of AKT signaling pathway‐related protein expression in CACO‐2 cells after different sample treatments for 24 h. (a) Expressions of AKT, p‐AKT(S473), and p‐AKT(T308) in CACO‐2 cells determined by Western blotting. (b and c) The relative density of each band was measured by ImageJ. (d–i) Results of significance analysis of the effect of combined treatment groups of PC and RES versus treatment groups alone on AKT signaling pathway‐related proteins in CACO‐2 cells.*p < .05, **p < .01, ***p < .001 compared to PC group. ^#^ *p* < .05, ^##^ *p* < .01, ^###^ *p* < .001 compared to RES group. ^ns^ *p* > .001 compared to PC or RES group.

AKT signaling pathway is associated with proliferation, differentiation, and apoptosis in cancer cells. In human solid tumors and hematologic malignancies, this is usually accompanied by excessive activation of the AKT signaling pathway (Manning & Toker, 2017). Therefore, the effect of PC combined with RES on AKT activity in CACO‐2 cells was explored. Western blotting showed that all three treatments were able to inhibit the phosphorylation of AKT protein (Figure 5a–c). When the sample concentration was 75 or 100 μM, the combined treatment group showed a stronger inhibitory effect on the phosphorylation of Thr308 protein than the separate treatment groups of PC and RES (Figure 5d–f). Under the three concentrations of 50, 75, and 100 μM, the combined treatment group resulted in significantly stronger inhibitory effect on phosphorylation of Ser473 protein was in than in the separate treatment group (Figure 5g‐i). This result suggested that PC and RES could synergistically inhibit the activation of AKT signaling pathway to some extent.

#### 3.5.2 ERK signaling pathway in CACO‐2 cells is synergistically inhibited by PC and RES

**FIGURE 6:**
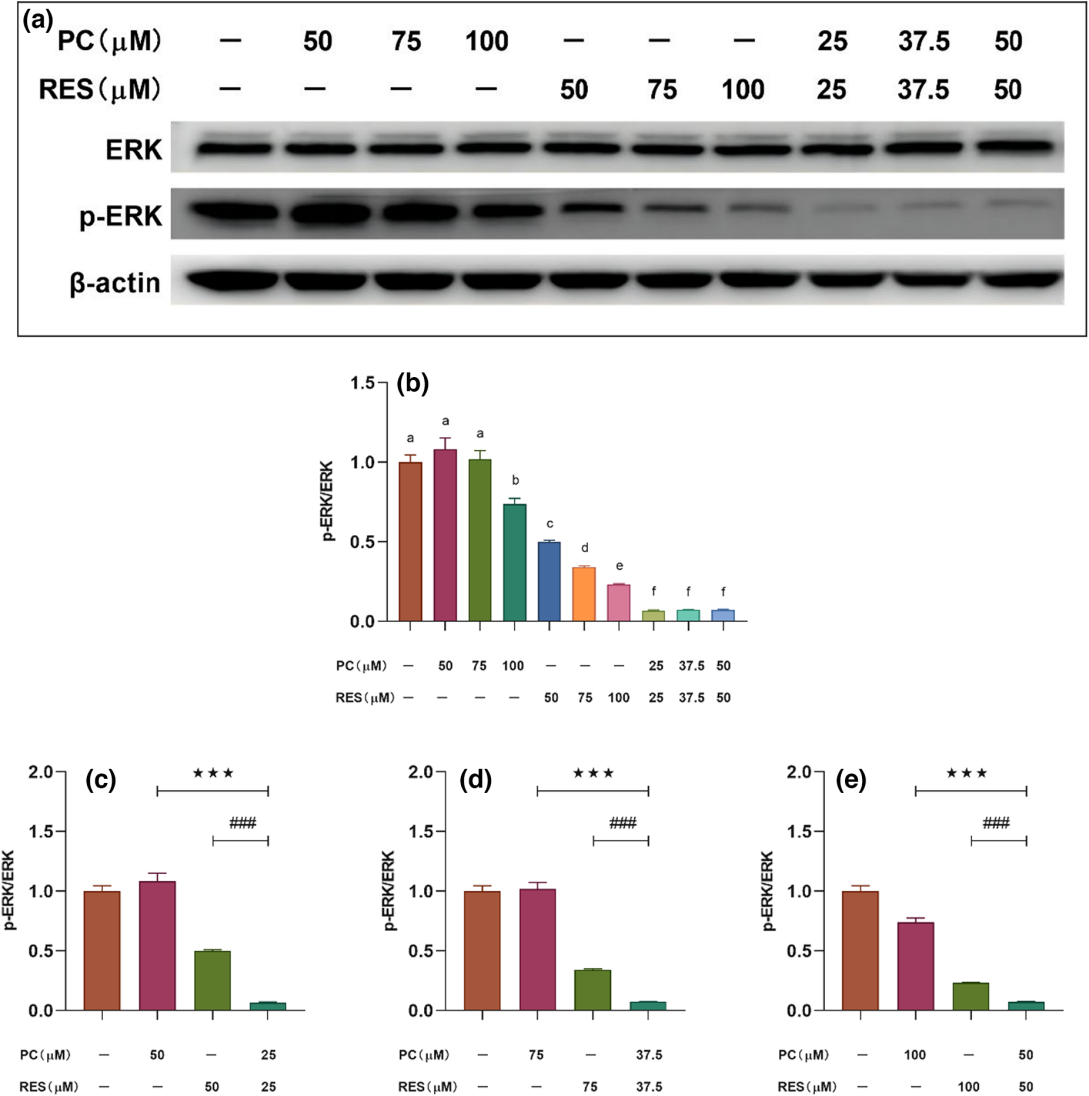
Changes of ERK signaling pathway‐related protein expression in CACO‐2 cells after different sample treatments for 24 h. (a) Expressions of ERK and p‐ERK in CACO‐2 cells determined by Western blotting. (b) The relative density of each band was measured by ImageJ. (c–e) Results of significance analysis of the effect of combined treatment groups of PC and RES versus treatment groups alone on ERK signaling pathway‐related proteins in CACO‐2 cells. ***p < .001 compared to PC group. ^###^ *p* < .001 compared to RES group.

ERK signaling pathway plays a key role in the pathogenesis and progression of human colorectal cancer. The effects of PC and RES on the activation of ERK signaling pathway in CACO‐2 cells were explored. The results were similar to their effects on PI3K/AKT signaling pathway. Phosphorylation of ERK protein was inhibited in CACO‐2 cells after treatment with these samples (Figure 6a,b), especially in the combined treatment group, in which the level of inhibition of ERK protein phosphorylation was significantly higher at these three concentrations than in the PC and RES treatment groups alone (Figure 6c–e).

#### 3.5.3 NF‐κB signaling pathway in CACO‐2 cells is synergistically activated by PC and RES

**FIGURE 7:**
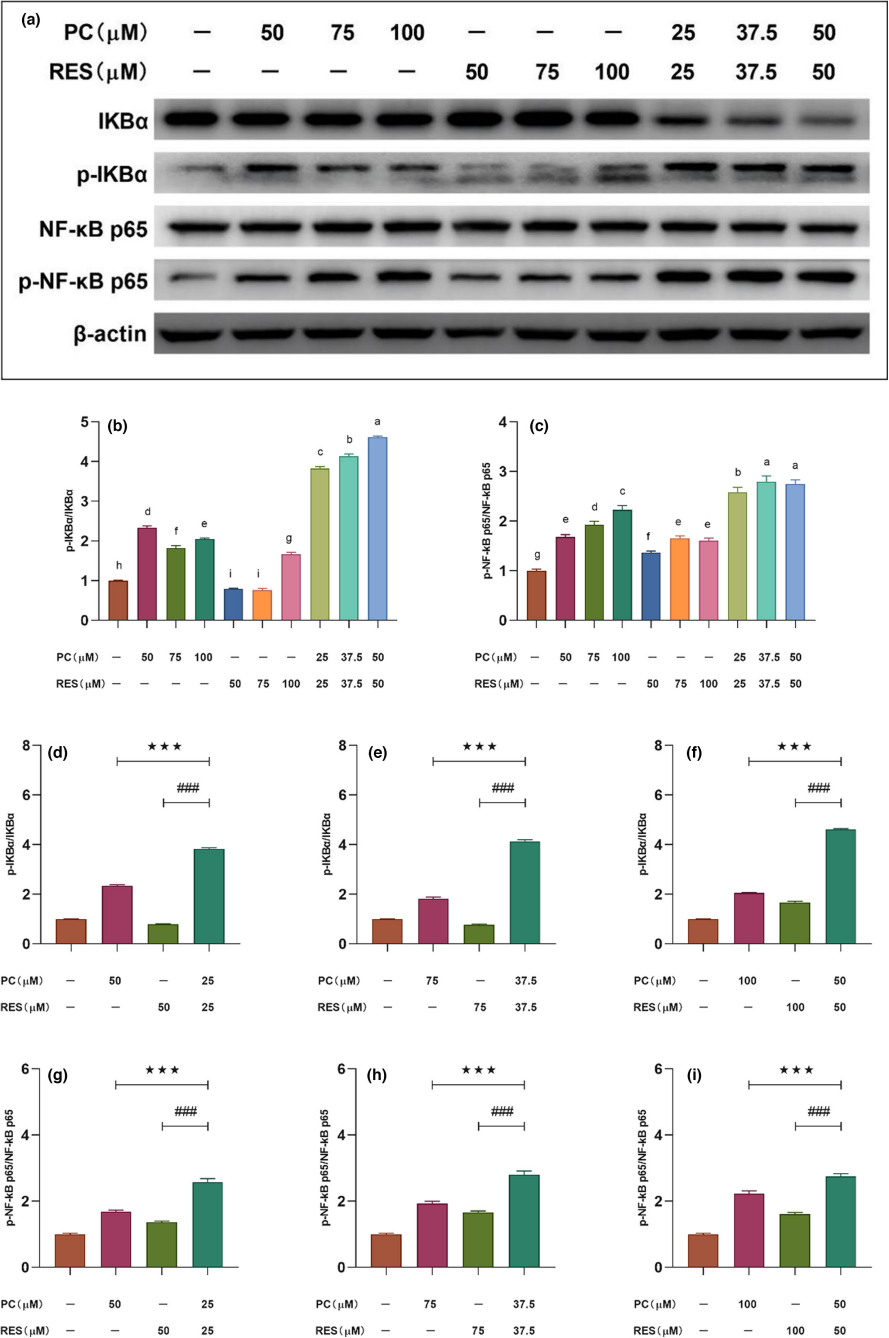
Changes of NF‐κB signaling pathway‐related protein expression in CACO‐2 cells after different sample treatments for 24 h. (a) Expressions of IKBα, p‐IKBα, NF‐κB p65, and p‐NF‐κB p65 in CACO‐2 cells determined by Western blotting. (b and c) The relative density of each band was measured by ImageJ. (d–i) Results of significance analysis of the effect of combined treatment groups of PC and RES versus treatment groups alone on NF‐κB signaling pathway‐related proteins in CACO‐2 cells. ***p < .001 compared to PC group. ^###^ *p* < .001 compared to RES group.

NF‐κB plays a role in inhibiting apoptosis in pathophysiological processes such as immunity, inflammation, tumor formation, and resistance of tumor cells to chemotherapeutic agents. However, in specific stimuli and in specific tumor cells, NF‐κB may also play a role in promoting apoptosis. Bian et al. showed that NF‐κB could mediate doxorubicin‐induced cell death in N‐type Neuroblastoma Cells (X. Bian et al., 2001), while Campbell et al. showed that NF‐κB could cause apoptosis by inhibiting the expression of anti‐apoptotic genes (Kirsteen et al., 2004). Similar to their findings, we found that the combined treatment group of PC and RES was able to promote the phosphorylation of IKBα and NF‐κB p65 proteins in CACO‐2 cells compared with the control group (Figure 7a–c), which represented that the NF‐κB signaling pathway was over‐activated. Moreover, the PC‐alone treatment group was also able to promote the phosphorylation of IKBα and NF‐κB p65 proteins at all three concentrations, but it was significantly less effective than the combined treatment group (Figure 7d–i). Notably, in the RES‐alone treatment group, phosphorylation of IKBα protein was inhibited only at a sample concentration of 100 μM, but phosphorylation of NF‐κB p65 protein was promoted in all concentrations.

These results explained that the growth‐inhibiting effect of PC and RES on CACO‐2 cells might be mediated by regulating the AKT, ERK, and NF‐κB signaling pathways.

## 4 DISCUSSION

In this study, we explored the effect of PC, RES, or their combined treatment for 24 h on the proliferation of CACO‐2, HCT‐8, HEPG‐2, and HUH‐7 cells using the CCK8 method. The present findings suggested that either PC or RES could inhibit the proliferation of these four cancer cells in a concentration‐dependent manner. However, PC and RES exhibited synergistic anticancer effects only on CACO‐2 colorectal cancer cells. Study revealed that multiple polyphenolic compounds are able to inhibit various cancer cell proliferations in different mechanisms. The combined effect of PC and RES on inhibiting the proliferation of CACO‐2 cells might be attributed to the simultaneous activation of multiple anticancer mechanisms. Additionally, cell clone formation assays were conducted in our study to evaluate the effect of PC combined with RES on the ability of CACO‐2 cells to grow and proliferate at a later stage. The results showed that PC and RES exhibited the same synergistic inhibitory effect in this regard. These experimental results provided preliminary evidence of the synergistic anticancer effect of PC and RES.

Apoptosis is an ordered cellular process and occurs under physiological and pathological conditions, playing a pivotal role in the pathogenesis of many diseases. However, cancer causes apoptosis to occur less frequently, thus impeding the death of malignant cells (Wong, 2011). Studies suggested that induction of apoptosis is an effective way to kill cancer cells. The detective results by flow cytometry found that PC could induce apoptosis in CACO‐2 cells alone or in combination with RES (Figure 4). The finding aligns with previous report that procyanidins synergistically induced apoptosis in breast cancer cells with resveratrol (Gao & Trygve, 2018). In addition, the effect of PC alone or in combination with RES on the cell cycle of CACO‐2 cells was investigated by flow cytometry. The results showed that they inhibited CACO‐2 cell proliferation by blocking the cell cycle in G0/G1 phase (Figure 3). These findings also validated the synergistic anticancer effects of PC and RES.

The serine/threonine protein kinase AKT (also known as protein kinase B) is activated in a variety of human tumors and is a central link in the classical PI3K‐AKT–mTOR signaling pathway. AKT plays a critical importance in regulating tumor cell growth and proliferation, promoting cell invasion and metastasis, promoting neovascularization, and in the development of resistance to chemotherapy and radiation therapy in tumor cells. Therefore, inhibiting tumor cell proliferation by the suppression of Akt activity is an effective approach to alleviate or treat cancer. Study found that procyanidins from grape seeds inactivated the PI3‐kinase/PKB pathway, inducing apoptosis in colon cancer cell lines (Engelbrecht et al., 2007). Liu et al. demonstrated that procyanidins B2 inhibited the proliferation of liver cancer cells by inhibiting AKT activity (G. Liu, Shi, Wang, Li, & Yin, 2020). The Western blot experiments in our study showed that phosphorylation of T308 decreased in CACO‐2 cells after treatment with PC. Unexpectedly, this phenomenon was more pronounced in the combined PC and RES treatment group, and phosphorylation of S473 was similarly reduced in the combined treatment group (Figure 5a–c). This experimental result suggested that apoptosis of CACO‐2 cells was induced by PC or PC combined with RES treatment accompanied by activation of AKT signaling pathway. In addition, there was a Cross‐Talk observed between the PI3K/AKT and RAS–ERK pathways. One pathway that was inhibited would often activate the other (Mendoza et al., 2011). One of the first substrates of AKT identified was the protein kinase c‐Raf (or Raf1), which was activated by RAS and initiated a kinase cascade culminating in ERK activation (Rommel et al., 1999). Therefore, it was speculated that the ERK signaling pathway might also be inhibited in apoptotic CACO‐2 cells after our sample treatment was speculated. Aligning with our conjecture, the ERK signaling pathway was similarly inhibited by PC or PC co‐RES in the results of Western blotting (Figure 5d,e).

NF‐κB is an important group of transcription factors involved in various physiological processes in cells and has complex regulatory mechanisms. Abnormal activation of the NF‐κB pathway can lead to abnormal expression of a cascade of tumor‐related genes. Therefore, there is no doubt that the NF‐κB family can promote tumor development as transcription factors. However, contradictory findings have also suggested that NF‐κB could also inhibit tumor development. Wu and others found that millepachine induced apoptosis by activating NF‐κB pathway in both SK‐OV‐3 and A2780S cells (Wu et al., 2016). The NF‐κB, especially its Rel (p65) subunit, as reported by Ryan et al., played an important role in p53‐mediated apoptosis‐inactivation or reduced activity of NF‐κB abolishes P53‐induced apoptosis (Ryan et al., 2000). In our study, treatment of CACO‐2 cells with PC alone or in combination with RES significantly promoted the phosphorylation of NF‐κB and IKBα, compared with other groups (Figure 5f–h). This result indicated that PC treatment of CACO‐2 cells activated the NF‐κB signaling pathway, and this effect could be amplified by the combined effect with RES.

Cancer cells have been shown to carry more ROS than normal cells (Szatrowski & Nathan, 1991). In the past few years, numerous studies had shown that reactive oxygen species play a crucial role in the survival of cancer cells. Edderkaoui et al. showed that ROS produced by the extracellular matrix increased the survival of pancreatic cancer cells through 5‐lipoxygenase and NOX (Edderkaoui et al., 2005). Lee et al. showed that ROS inhibited protein tyrosine phosphatase, thereby maintaining kinase‐mediated activation of the anti‐apoptotic pathway in pancreatic cancer cells activation in pancreatic cancer cells (Lee et al., 2007). Similarly, the role of ROS in tumor cell proliferation had been reported in many experiments. For example, treatment of tumor cells with low concentrations of exogenous H_2_O_2_ increased intracellular ROS levels and promoted cell proliferation (Kim et al., 2012; Wang et al., 2013). Hemoglobin induced colon cancer cell proliferation by releasing ROS (Lee et al., 2006). The proliferation of cancer cells was prevented by inhibiting Lon molecules, an inducer of ROS in bladder cancer cells (Liu et al., 2014). In our study, the constituents of PC and RES were performed by extensive spectrometry analysis. The results showed that procyanidin A was the main component of PC extract (Wang et al., 2022), and the main components of RES extract were oxidized resveratrol, resveratrol glycosides, resveratrol dimer, and tetramer (Appendix S1). We speculated that the inhibitory effect of PC on the growth and proliferation of CACO‐2 colorectal cancer cells might attribute to the stronger antioxidant capacity of procyanidin A but the result of which needs to be further verified. It is suggested that the synergistic anticancer effect of PC and RES may be related to these components, and the effect of specific components needs further study.

## 5 CONCLUSION

In this study, we found that both PC and RES synergistically inhibited the growth and proliferation of human colorectal cancer cells (CACO‐2, HCT‐8) and liver cancer cells (HEPG‐2, HUH‐7) in a concentration‐dependent manner. PC and RES showed a positive synergistic trend in inhibiting the growth of CACO‐2 colorectal cancer cells, which was also further verified in our cell clone formation assay, cell cycle assay, and apoptosis assay. Additionally, Western blot results showed that PC combined with RES may exert its synergistic anticancer effects by inhibiting AKT and ERK signaling pathways and promoting NF‐κB signaling pathway. Among these four cells, PC and RES produced synergistic anticancer effects only on CACO‐2 colorectal cancer cells, which provide new ideas for precise preventive interventions of cancer and new insights for effective treatment of colorectal cancer.

## AUTHOR CONTRIBUTIONS

**Na Wang:** Conceptualization (equal); investigation (equal); methodology (equal); project administration (equal); resources (equal); supervision (equal); writing – original draft (equal); writing – review and editing (equal). **Enguang Gao:** Conceptualization (equal); data curation (equal); methodology (equal); project administration (equal); software (equal); writing – original draft (equal); writing – review and editing (equal). **Chenxu Cui:** Methodology (equal). **Fan Wang:** Writing – review and editing (equal). **Hongtao Ren:** Writing – review and editing (equal). **Chao Xu:** Data curation (equal). **Cancan Ning:** Writing – review and editing (equal). **Yuru Zheng:** Data curation (equal). **Qingqing Liu:** Formal analysis (equal). **Qiuying Yu:** Formal analysis (equal); methodology (equal); writing – original draft (equal); writing – review and editing (equal). **Gaiping Zhang:** Conceptualization (equal); investigation (equal); methodology (equal); resources (equal).

## FUNDING INFORMATION

The research work was not funded by any project.

## CONFLICT OF INTEREST STATEMENT

The authors declare no conflicts of interest.

## CONSENT

All authors agreed for the publication of this manuscript.

## Supporting information


Appendix S1.

## Data Availability

The data that support the findings of this study are available from the corresponding author, [NW], upon reasonable request.
