# Supplementary material for: The combined anticancer of peanut skin procyanidins and resveratrol to CACO‐2 colorectal cancer cells
Source: Food Sci Nutr. 2023 Aug 3;11(10):6483–97. doi: 10.1002/fsn3.3590 (PMC10563709; doi:10.1002/fsn3.3590)

**Extended Data**

**Composition of Peanut Bud Resveratrol (Res)**

**Figure legends:**

**Figure S1** Total negative ion chromatogram of Res.

**Figure S2** MS/MS spectra of Res compounds in negative ion mode.

**Table legends:**

**Table S1** Mass spectrometry information of the main compounds in Res.

**Figure S1**


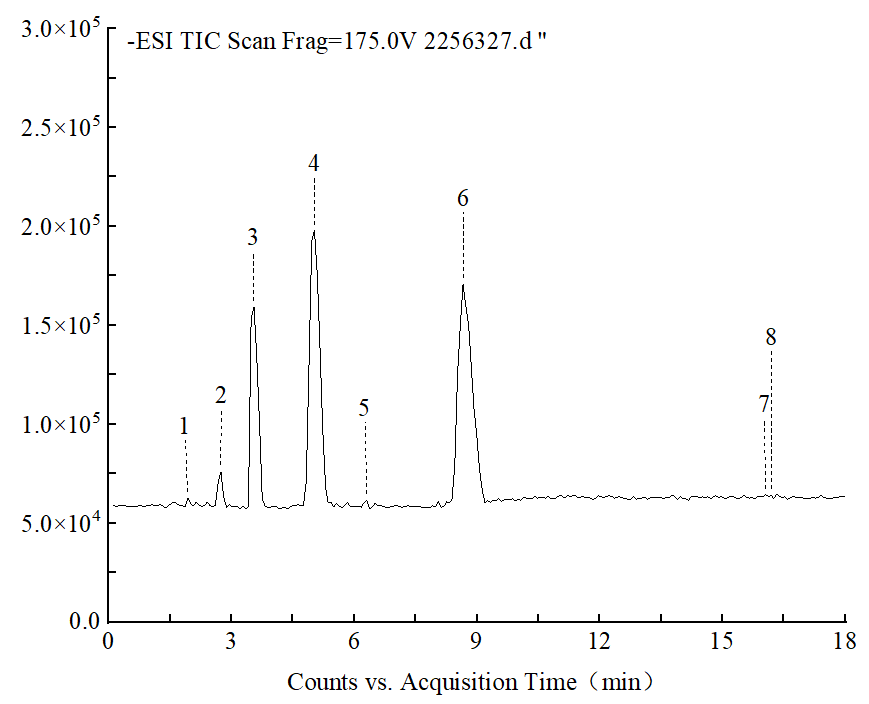


**Table S1**

| Serial number | Test substance | Retention time /min | [M-H]-（m/z） | Fragment ions（m/z） |
| --- | --- | --- | --- | --- |
| 1 | A-type proanthocyanidin trimer | 1.898 | 863 | 711.13、575.12、451.08、411.07、289.06 |
| 2 | A-type proanthocyanidin trimer | 2.781 | 862 | 711.08、575.15、451.10 |
| 3 | A-type proanthocyanidin dimer | 3.596 | 575 | 289.07、285.06 |
| 4 | Resveratrol oxide | 4.933 | 243 | 201.10、159.09 |
| 5 | Resveratrol | 6.321 | 227 | 185.06、158.98、143.15、114.09 |
| 6 | Epigallocatechin gallate | 8.768 | 441 | 288.42、168.92 |
| 7 | Resveratrol dimer | 16.033 | 451 | 435.02、409.18、305.91 |
| 8 | Resveratrol glycoside | 16.176 | 389 | 185.00、143.21 |

**Figure S2**


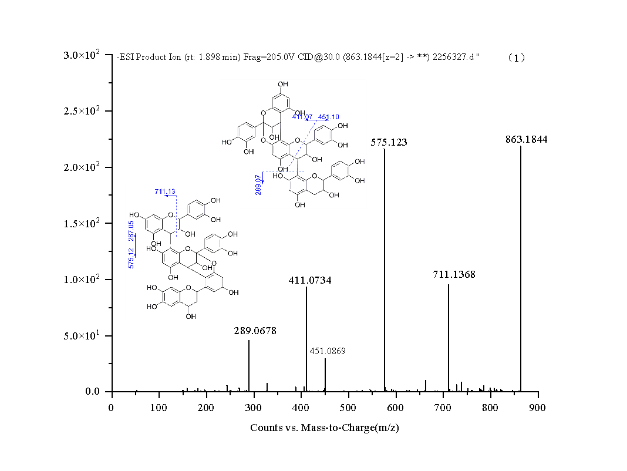

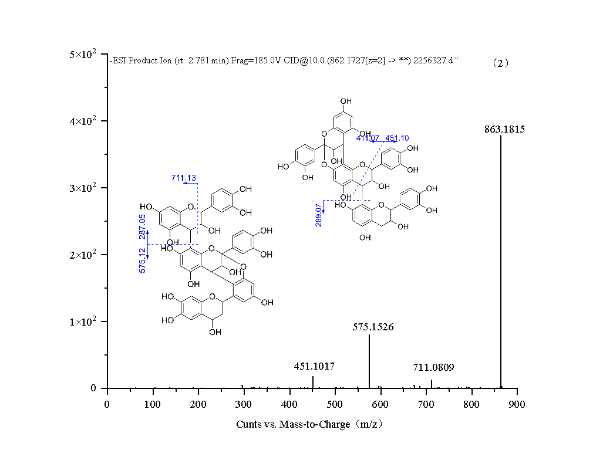


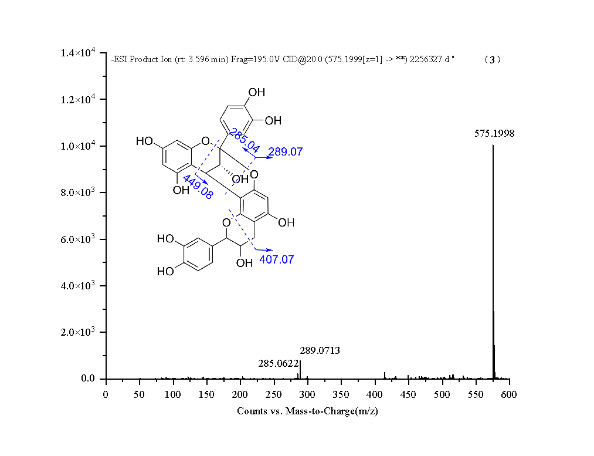

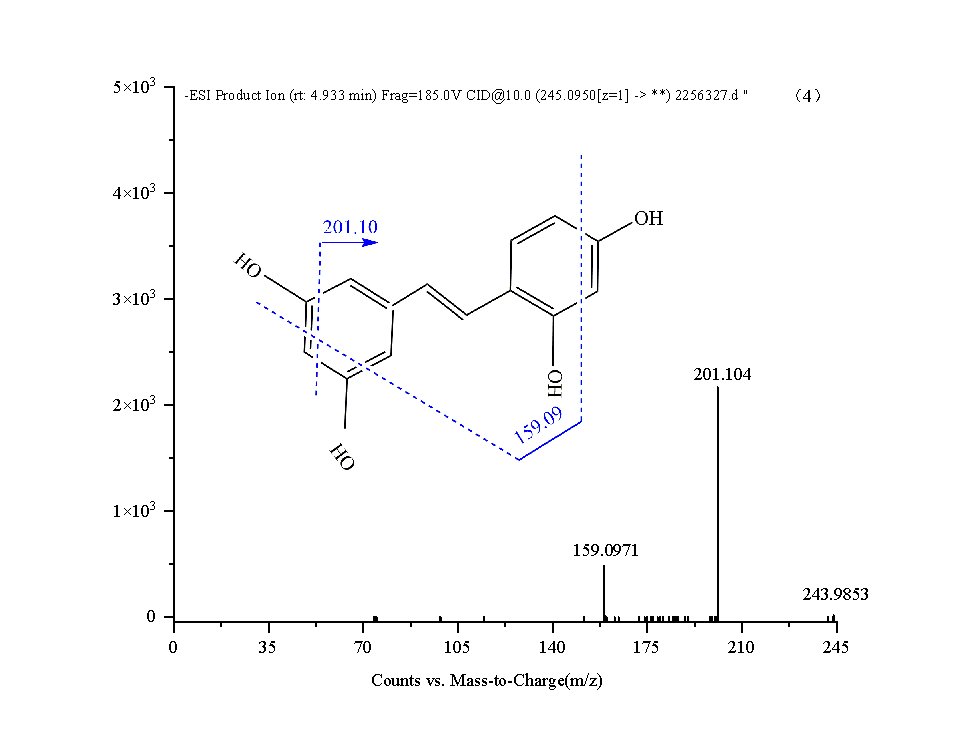


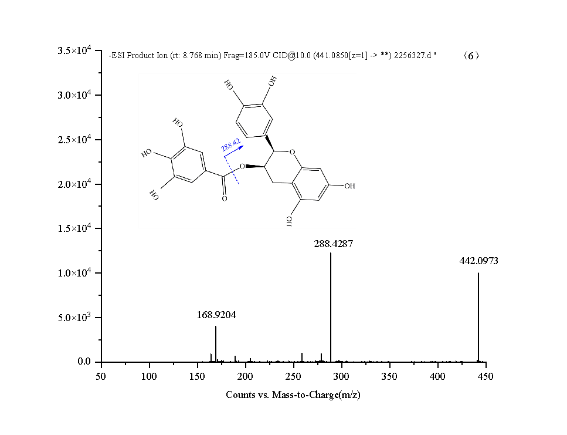

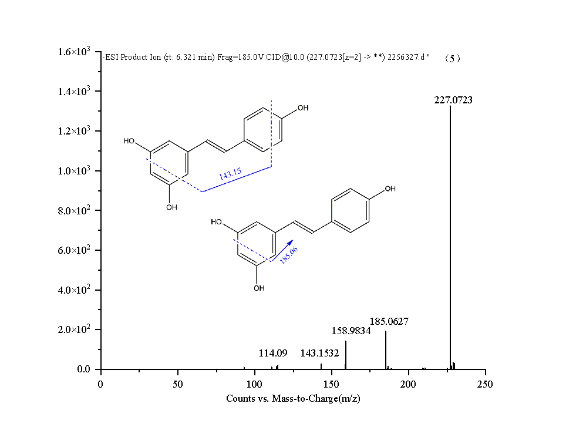

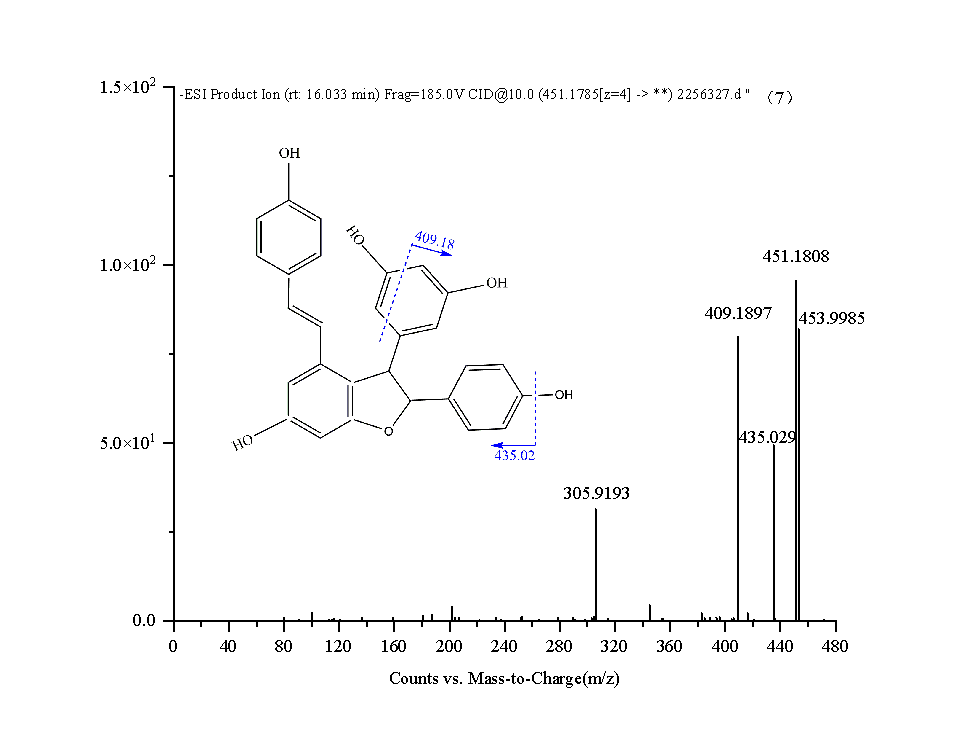

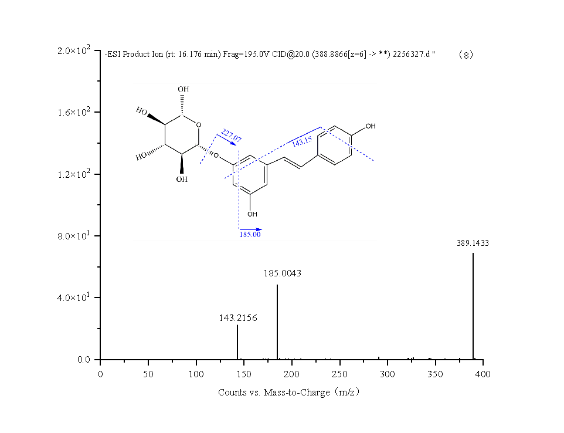

Supplement: Supplementary file 1 — Appendix S1. [file FSN3-11-6483-s001.docx]
